# UPLC/MS-based untargeted metabolomics reveals the changes of metabolites profile of *Salvia miltiorrhiza* bunge during *Sweating* processing

**DOI:** 10.1038/s41598-020-76650-w

**Published:** 2020-11-11

**Authors:** Mengyang Cao, Yingying Liu, Weimin Jiang, Xiaoxi Meng, Wei Zhang, Weidong Chen, Daiyin Peng, Shihai Xing

**Affiliations:** 1 College of Pharmacy, Anhui University of Chinese Medicine, Room 405, Qianjiang Road No. 1, Yaohai Disrict, Hefei, 230012 Anhui Province China; 2 Institute of Traditional Chinese Medicine Resources Protection and Development, Anhui Academy of Chinese Medicine, Hefei, 230012 China; 3 College of Humanities and International Education Exchange, Anhui University of Chinese Medicine, Hefei, 230012 China; 4 College of Life Sciences and Environment, Hengyang Normal University, Hengyang, 421008 Hunan China; 5 Department of Horticultural Science, University of Minnesota, Minneapolis, MN 55108 USA; 6 Synergetic Innovation Center of Anhui Authentic Chinese Medicine Quality Improvement, Hefei, 230038 China; 7 Anhui Province Key Laboratory of Research and Development of Chinese Medicine, Hefei, 230012 China

**Keywords:** Biological techniques, Biotechnology, Chemical biology, Plant sciences

## Abstract

*Salvia miltiorrhiza* has numerous compounds with extensive clinical application. “*Sweating*”, a processing method of Traditional Chinese Medicine (TCM), results in great changes in pharmacology and pharmacodynamics. Previously, chromatogram of 10 characteristic metabolites in *S*. *miltiorrhiza* showed a significant difference after “*Sweating*”. Due to the complexity of TCM, changes in metabolites should be investigated metabolome-wide after “*Sweating*”. An untargeted UPLC/MS-based metabolomics was performed to discover metabolites profile variation of *S*. *miltiorrhiza* after “*Sweating*”. Multivariate analysis was conducted to screen differential metabolites. Analysis indicated distinct differences between sweated and non-sweated samples. 10,108 substance peaks had been detected altogether, and 4759 metabolites had been identified from negative and positive ion model. 287 differential metabolites were screened including 112 up-regulated and 175 down-regulated and they belong to lipids and lipid-like molecules, and phenylpropanoid and polyketides. KEGG analysis showed the pathway of linoleic acid metabolism, and glyoxylate and dicarboxylate metabolism were mainly enriched. 31 and 49 identified metabolites were exclusively detected in SSM and NSSM, respectively, which mainly belong to carboxylic acids and derivatives, polyketides and fatty acyls. By mapping tanshinones and salvianolic acids to 4759 identified metabolites library, 23 characteristic metabolites had been identified, among which 11 metabolites changed most. We conclude that *“Sweating’’* has significant effect on metabolites content and composition of *S. miltiorrhiza*.

## Introduction

*Salvia miltiorrhiza* Bunge, referred to as “Danshen” in China or “Tanshen” in Japan, which is an important species in Salvia genus with great medicinal values^1^, was first recorded in *Shennong Bencao Jing*, the earliest famous extant monograph of traditional Chinese pharmacology in China^2^. The dry roots and rhizomes of *S*. *miltiorrhiza*, called Danshen, have been extensively used in the clinical treatments of many different diseases, such as coronary heart disease^3^, hyperlipidemia^4^, cerebrovascular disease^5^, Alzheimer’s disease^6^, hypertension^7^, cancer^8^ and osteoporosis^9^.

Because Danshen has a broad prospect of clinical application, lots of effort has been made to investigate the main constituents and bioactive ingredients of Danshen^10^. The bioactive components of Danshen are mainly divided into fat-soluble tanshinones belonging to diterpene and water-soluble caffeic acid-derived salvianolic acids^11,12^. Thus far, more than 40 tanshinones and over 50 salvianolic acids have been well isolated and identified from roots of *S*. *miltiorrhiza*. Tanshinones mainly include tanshinone I, tanshinone IIA (TsIIA), tanshinone IIB, cryptotanshinone and dihydrotanshinone I, and salvianolic acids mainly consist of salvianolic acid A (Sal A), salvianolic acid B (Sal B), lithospermic acid, danshensu, caffeic acid, and rosmarinic acid^13,14^.

The initial processing of TCM is important and closely related to the quality of TCM’s products^22^. The initial processing of medicinal materials is from the harvest of medicinal parts to the formation of commercial medicinal materials, such as preliminary treatment and drying^23^. At present, initial processing methods mainly include slicing while the herbs are still fresh, drying and packing^24^. Medicinal materials are formed through the above-mentioned processing, during which physical and chemical changes take place, such as the removal of non-medicinal parts, the maximum retention of active substances, the reduction of toxic components, and the mutual transformation of chemical components^23^. The medicinal materials forming during initial processing are the raw materials of Chinese herbal slices. Their quality directly affects the quality of Chinese herbal slices, and then affects the clinical curative effect of TCM^25^.

“*Sweating*” is the main initial processing method for Danshen roughly as follows: the fresh roots of *S*. *miltiorrhiza* are piled up and covered to produce heat and emit moisture to the surface of the materials^26^. “*Sweating*” is beneficial not only to the drying of herbs, but also to regulate and promote the enzyme system and microbial community activity in herbs tissues, and accelerate the process of biotransformation and chemical transformation of primary and secondary metabolites, which directly affects the quality of medicinal materials^27^. Pharmacological activities of medicinal materials can be changed by “*Sweating*”. Some related pharmacodynamic studies have shown that the antioxidant activity^28^, brain protective effect, and myocardial protective effect of “*Sweating*” *S*. *miltiorrhiza* (SSM) are stronger than those of non-sweating *S*. *miltiorrhiza* (NSSM)^29^. Cocktail probed drug method has been used to study the effects of SSM and NSSM on CYP450 enzyme activity in rats. The result has shown that there are significant differences in effects of SSM and NSSM on CYP450 enzyme activity in rats^28^. The unique pharmacology and efficacy of SSM is a reflection of hundreds of bioactive compounds rather than a single or several ones. In addition, the various activities of components in Danshen are considered responsible for the therapeutic effects of Danshen^30^. Therefore, it is necessary to study the changes of the composition and content of chemical components in roots of *S*. *miltiorrhiza* after “*Sweating*”. However, due to the complexity of Traditional Chinese Medicine (TCM), it’s far from enough to be merely focused on several main bioactive components in TCM but ignore the whole changes of the metabolites^31^, and it’s difficult to comprehensively and accurately investigate the effect of “*Sweating*” on the chemical composition of Danshen by only determining some main components*.* Consequently, studies of the overall chemicals profile of “*Sweating*” on Danshen are necessary to better understand the mechanism of “*Sweating*”.

Untargeted metabolomics is a valuable technique for comprehensive analysis and comparison of end-product metabolites in the process of cell regulatory processes, and their levels can be considered as the ultimate response of the biological system to changes in endogenous and/or exogenous factors^32^. The LC–MS method is a useful tool to analyze the components of herbal medicine, which provides efficient visualization and identification of the metabolites^33^ It shows metabolite selectivity and sensitivity to targeted analysis, and detects low concentration compounds that may not be detected by other methods^34^. In this study, we used *S*. *miltiorrhiza* without “*Sweating*” (NSSM) and Danshen just after “*Sweating*” (SSM) as experimental materials. UPLC-MS-based metabolomics combined with multivariate statistical analysis was applied to reveal the metabolic profile differences between NSSM and SSM so as to investigate the effect of “*Sweating*” on the global metabolic profile of Danshen.

The difference of components between SSM and NSSM materials in this study merely comes from “*Sweating*” process without long-term storage leading to complex components loss, with which may result in non-unique difference. Additionally, we compared the changes of some main characteristic bioactive components in Danshen before and after “S*weating*” to specifically reveal the effect of “*Sweating*”. The results gave a deeper insight into the mechanism behind “*Sweating*” of Danshen and a better explanation of the scientific connotation of “*Sweating*”.

## Results and discussion

### Identification of global metabolite profiling

Methanol extracts from the roots of SSMs and NSSMs were measured by a UPLC system fitted with Q-Exactive quadrupole-Orbitrap mass spectrometer (Thermo Fisher Scientific, Waltham, MA, USA) to investigate the metabolic changes during the “*Sweating*” processing and identify critical metabolites responsible for metabolomics variation by “*Sweating*” processing.

**Figure 1:**
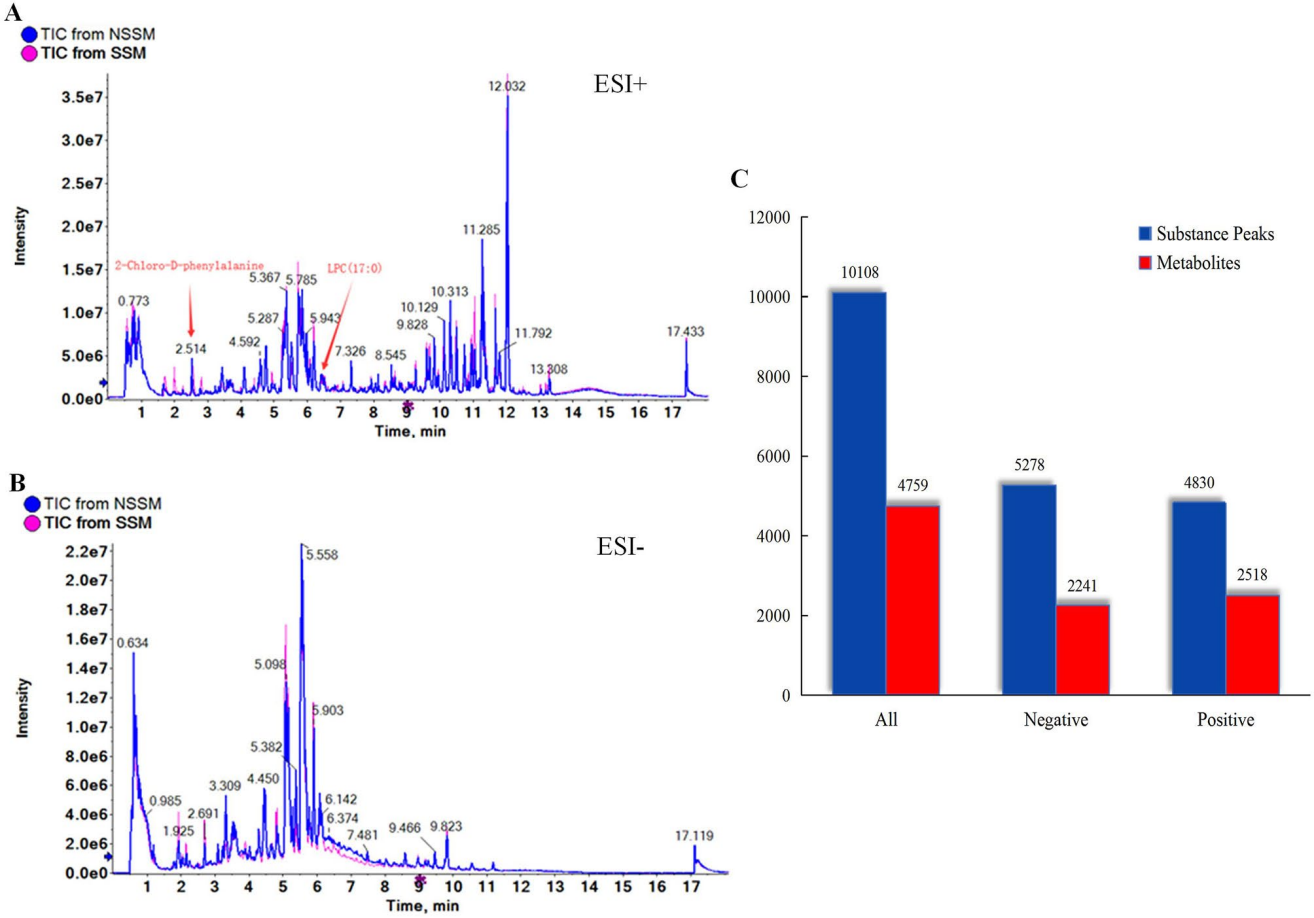
Total ions chromatograms (TICs) of methanol extracts from SSM and NSSM and global metabolites identified. **a** TICs of positive electrospray ionization (ESI^+^), and arrows indicate inner standards, **b** TICs of negative ESI (ESI^-^), **c** The number of substance peaks determined and metabolites identified.

Typical total ions current chromatograms (TICs) of SSM and NSSM is shown in Fig. 1a (ESI^+^) and Fig. 1b (ESI^-^), respectively. L-2-chlorophenylalanine (C_9_H_10_ClNO_2_) with RT (retention time) at 2.514 min and LPC (17: 0) (C_25_H_52_NO_7_P) with RT 6.430 min was added to the extracts as two internal standards (L-2-chlorophenylalanine with mass number 200.0478, 0.3 mg/mL; Lyso PC17: 0 with mass number 510.3481, 0.3 mg/mL). Metabolites in Danshen samples were identified according to internal standards and metabolomics databases of the Human Metabolome Database (HMDB), Lipidmaps (v 2.3) and METLIN by the software of Progenesis QI V2.3. A total of 10,108 substance peaks were detected by UPLC-MS in the samples of SSM and NSSM, among which 4759 metabolites were identified including 2241 metabolites from negative ion model (ESI^-^) and 2518 metabolites from positive ion mode (ESI^+^), respectively (Fig. 1c, Supplementary Table S1).

### Multivariate analysis of identified metabolites

**Figure 2:**
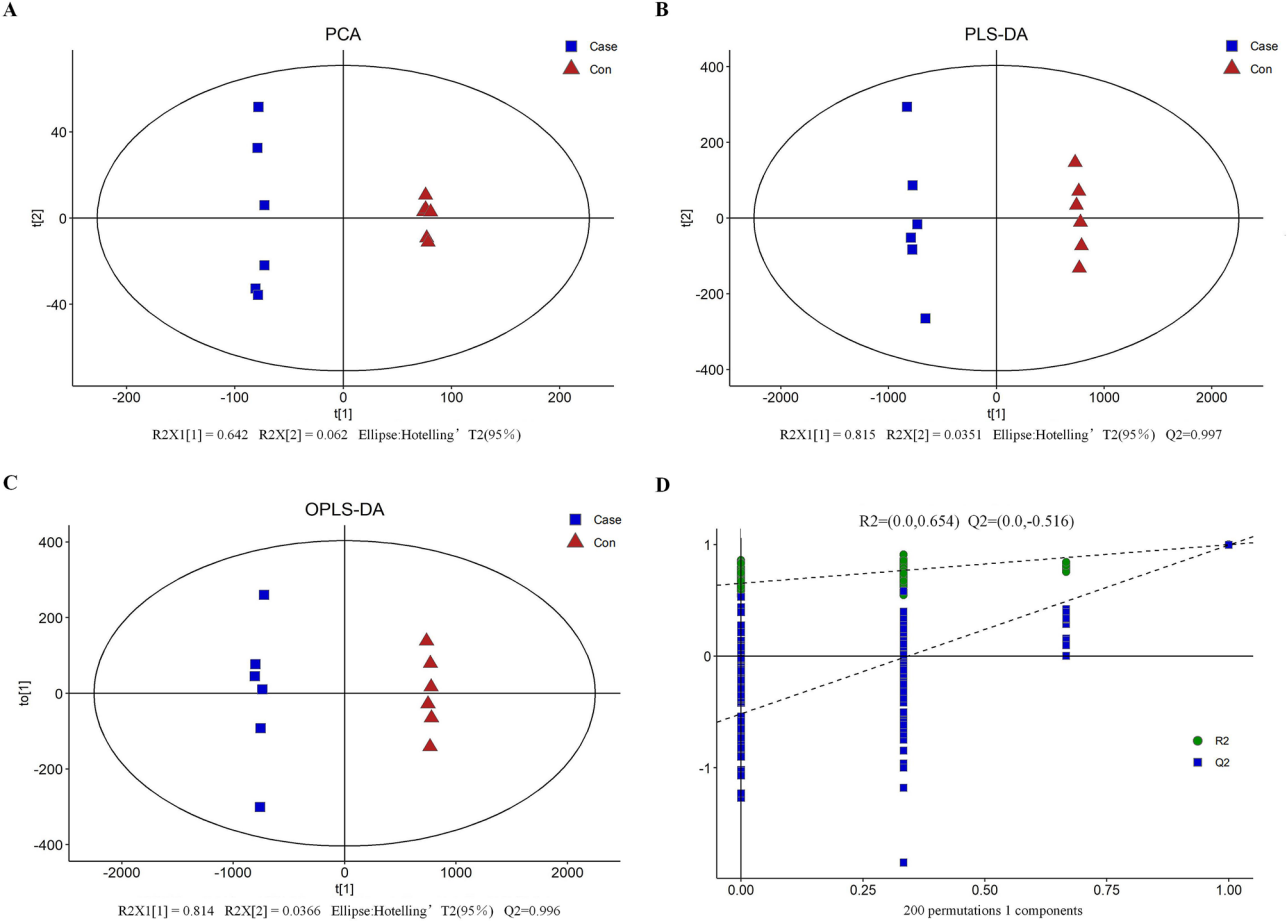
Model of multivariate analysis and its cross validation. **a** Principal components analysis (PCA), **b** partial least square discrimination analysis (PLS-DA), **c** orthogonal PLS-DA (OPLS-DA), **d** response permutation testing of the model predicted by OPLS-DA. R2X (cum): cumulative interpretation rate in X direction, R2Y (cum): cumulative interpretation rate in Y direction, Q2 (cum): cumulative forecast rate of model, R2 and Q2: parameters of response sequencing test, used to measure whether the model is over fitted.

Unsupervised principal components analysis (PCA) was carried out on the detected 10,108 substance peaks, QC samples and the two kinds of samples SSM and NSSM were classified into different metabolite profile with a significance of 0.05 by PCA, showing good stability and repeatability of the LC/MS detection (Fig. 2a). In order to visualize the metabolites differences between experimental samples and QC samples, and eliminate the effects of quantity on pattern recognition, hierarchical cluster analysis (HCA) and a log10 transformation of peak areas for each metabolite were performed. Samples of SSM, NSSM and QC were merged into independent cluster (Supplementary Figure S1). Thus, PCA combined with HCA indicates that samples of SSM and NSSM had remarkably differential metabolic profiles.

The supervised method, partial least square discrimination analysis (PLS-DA) was also applied to investigate the metabolites difference between SSM and NSSM samples (Fig. 2b). The calculated R^2^ value of PCA and Q value of PLS-DA are shown in Fig. 2, and the values indicated that there was statistically significant difference between SSM and NSSM. The orthogonal PLS-DA (OPLS-DA), another supervised method, was used to highlight the quantitative variation in the metabolites between two samples, and good model quality was also shown by the value of R^2^X and Q^2^ (Fig. 2c). The OPLS-DA score of each sample in this figure once again showed that there was significant difference among them (p < 0.5). The cross-validation with 200 permutation tests indicated that this OPLS-DA model was reliable, with intercepts of R^2^ and Q^2^ equal to 0.654 and − 0.516, respectively (Fig. 2d).

### Screening and classification of the differential metabolites

**Figure 3:**
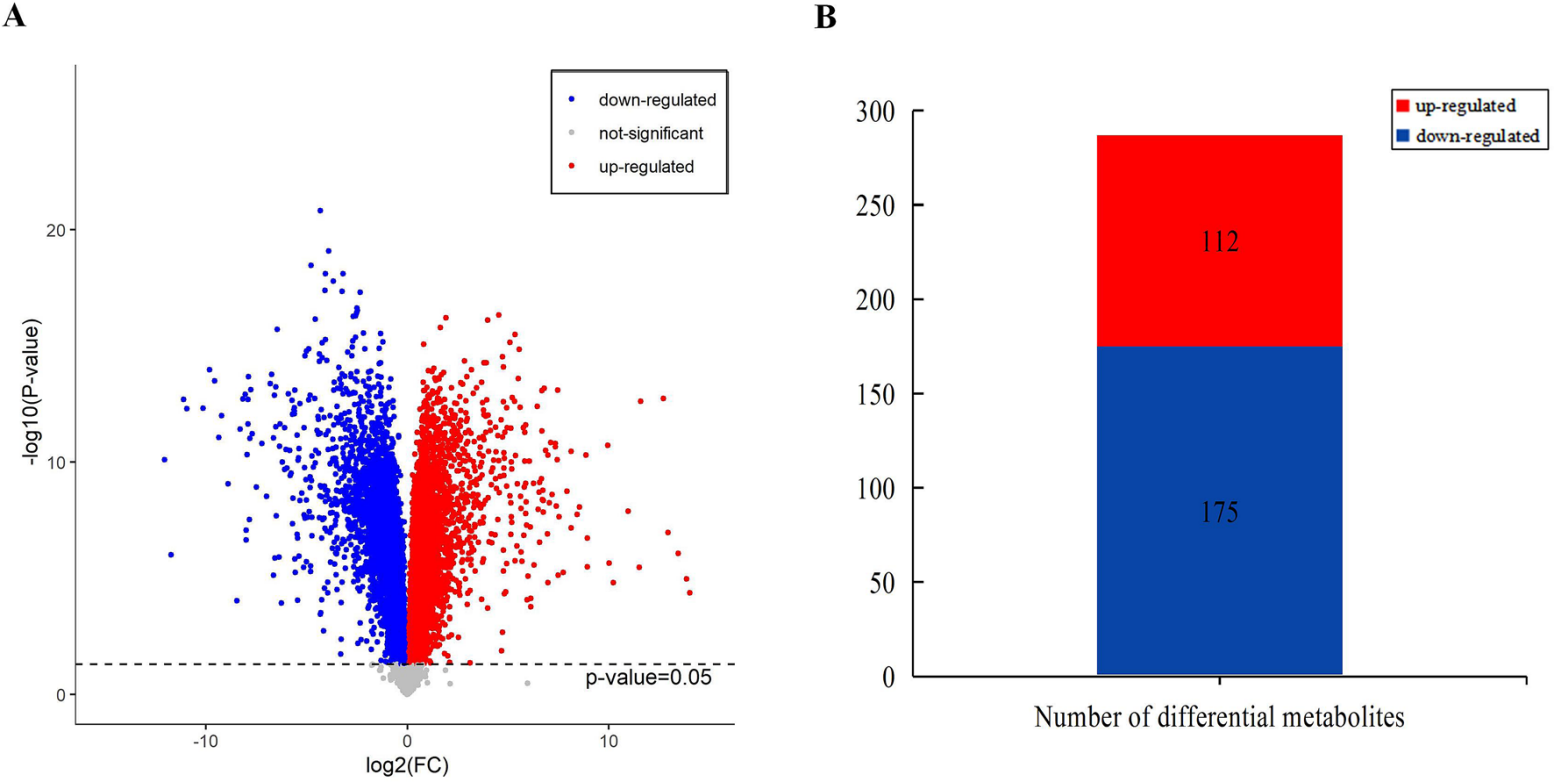
Differentially accumulating metabolites between SSM and NSSM. **a** Differential gene volcano plot of the 4759 metabolites identified. Differential metabolites were defined as metabolites with fold change ≥ 1.6 or ≤ 0.625 in SSM compared with NSSM. A threshold of VIP > 1.0 was used to separate differential metabolites from not-significantly differential metabolites, **b** number of differential metabolites between SSM and NSSM.

To visualize the result of fold change analysis of 10,108 substance peaks, a volcano plot was drawn by transferring the fold change (FC) value of each substance peak to log2 (FC), and transferring the P value (P = 0.05) of Student’s t test to –log10(P value). Among all of the differential metabolites, upregulated metabolites (SSM compared with NSSM) were symbolized with red dots gathered into a group and downregulated metabolites with blue dots gathered into a group (Fig. 3a). As shown in the volcano plot, there were obvious differences in metabolites between the two samples.

To identify differential metabolites between samples, a fold change ≥ 1.6 (up-regulated) or ≤ 0.625 (down-regulated) in SSM compared to NSSM, and these metabolites were screened using a variable importance in projection (VIP) value (VIP ≥ 1.0) from the OPLS-DA model. A total of 287 differential metabolites had been identified from total 4759 identified metabolites between SSM and NSSM by VIP and FC value, containing 130 and 157 metabolites from the positive and negative model, respectively (Fig. 3b, Supplementary Table S2).

**Figure 4:**
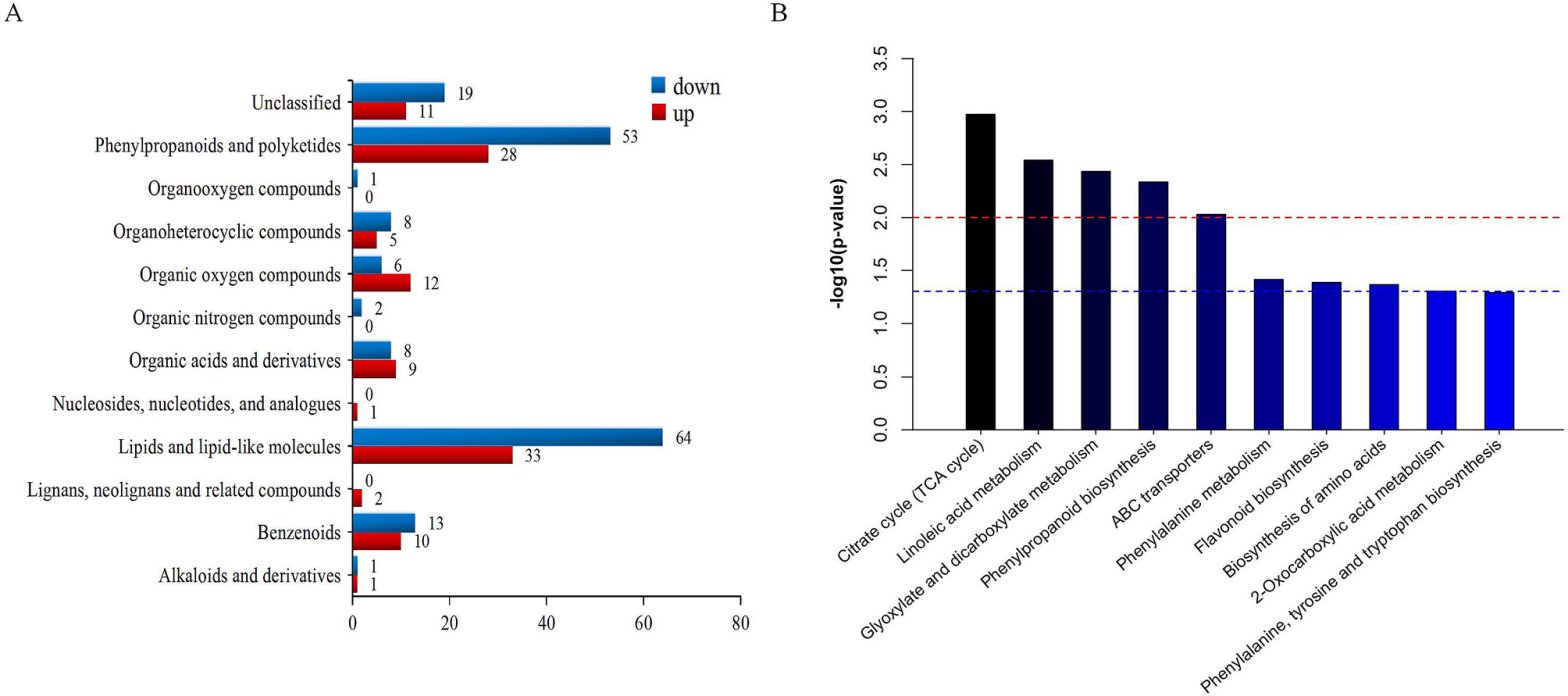
Classification of differential metabolites and pathways enrichment. **a** Classification of differential metabolites, **b** top 10 enriched pathways, red line dotted line shows *P*-value is 0.01 and blue dotted line shows *P*-value is 0.05.

Of these differential metabolites, 112 metabolites were up-regulated and 175 metabolites were down-regulated in SSM compared with NSSM. The majority of differential metabolites were lipids and lipid-like molecules, phenylpropanoid and polyketides, benzenoids, organic acids and derivatives, organic oxygen compounds, and there were 30 unclassified chemicals (Fig. 4a). These results showed the process of “*Sweating*” notably changed the metabolic profile of Danshen.

Emerging literature has indicated that lipids and lipid-like molecules have less anti-inflammatory properties, higher antioxidant effects, immunomodulatory effect, and decrease the risk of cholestasis, and improve clinical outcomes of certain subgroups of patients^35^. Research has shown that phenylpropanoid and polyketides possess anticarcinogenic, anti-inflammatory, neuroprotective, immunomodulatory, antihypertensive, and metabolic effects^36^. Benzenoids have the effect on anti-inflammatory^37^. All of these results indicate that changes in these differential metabolites are responsible for great change in Danshen’s pharmacological effects during the process of “*Sweating*”.

### KEGG classification and enrichment analysis of differential metabolites

We mapped the 287 differential metabolites to the KEGG (Kyoto Encyclopedia of Genes and Genomes) database, looking first at information about pathways^38^. Our result showed that 51 metabolites mapped to “metabolism” and they had KEGG ID. Subsequently, KEGG pathway enrichment analysis was conducted to identify differences in metabolic pathway between SSM and NSSM samples. 19 metabolites had been found enriched in 30 KEGG pathways (Supplementary Table 3). By calculating the –lg(*P*-value) of each pathway, linoleic acid metabolism was the most enriched pathway, glyoxylate and dicarboxylate metabolism and phenylpropanoid biosynthesis were also the main enriched pathways (Fig. 4b).

Previous study indicated that linoleic acid and related metabolic pathways are associated with bone mineral density^39^. Linoleic acid help to protect liver safety in obese and overweight women and fight against mortality^40,41^. Study also found biomarkers of *S. miltiorrhiza* that can improve hemorheological disorder and vascular endothelial function of microcirculation dysfunction rats are mainly related to linoleic acid metabolism, glutathione metabolism, and glyoxylate and dicarboxylate metabolism etc. All of the research indicated that changes in the core metabolites and their corresponding pathways culminate in improvement on pharmacological function of Danshen after “*Sweating*”.

### Top 50 differential metabolites analysis

**Figure 5:**
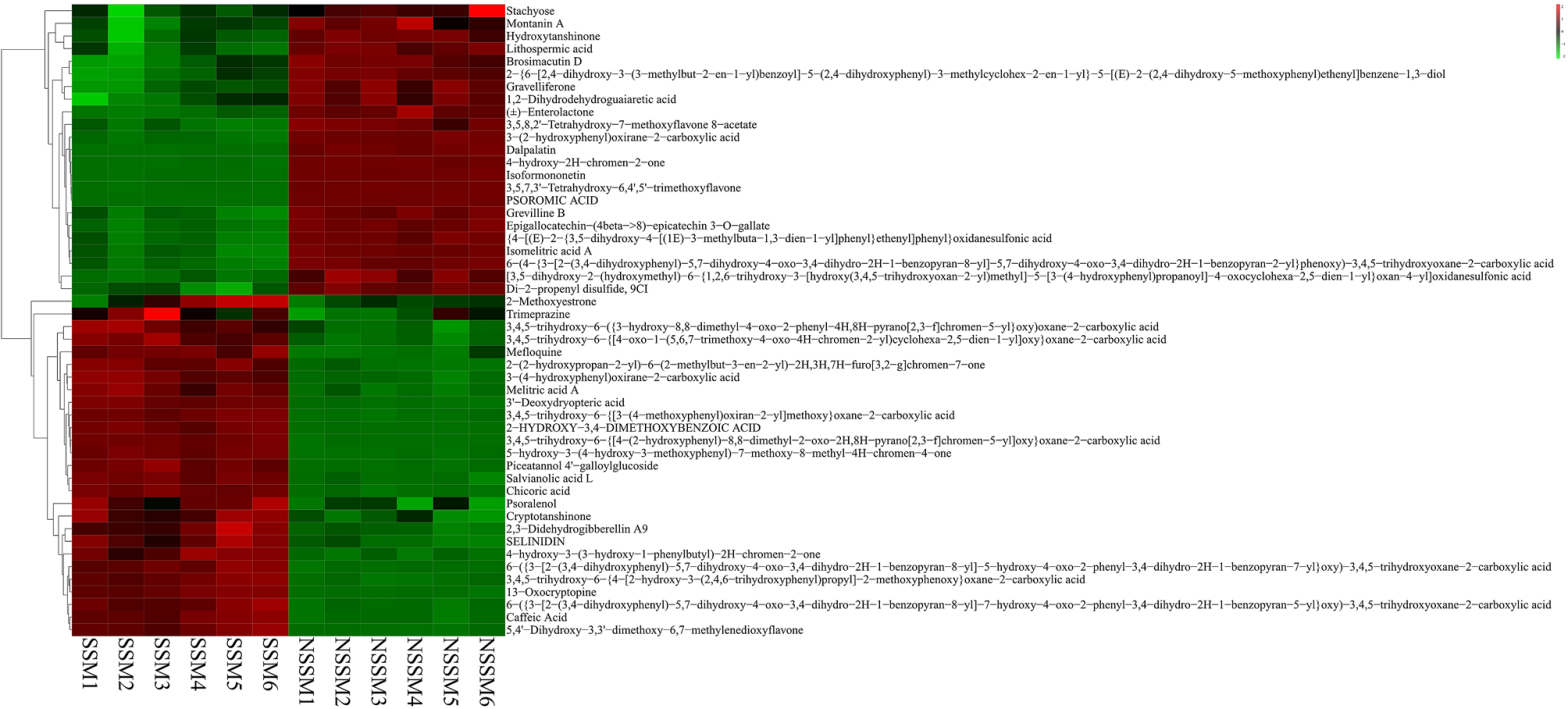
Heatmap of top 50 differential metabolites.

Among these 297 differential metabolites, those differential metabolites in top 50 VIP value were collected. Ranking these metabolites by FC calculated from data matrix, it showed that the content of indoleacrylic acid with the highest content by “*Sweating*” was at 7.54 folds, while the content of psoromic acid was only about 5% compared to NSSM (Supplementary Table S4). A heatmap was drawn using the top 50 differential metabolites, there’s significant difference between SSM and NSSM materials (Fig. 5). The conclusion can be reached that there was a marked change in the content of compounds during Danshen *“Sweating”* processing.

Indoleacrylic acid (IAA), as a plant hormone, was found to have the function to promote anti-inflammatory responses and therapeutic benefits^42^, and previous study has shown that IAA can inhibit tumor cell invasion, migration and cell proliferation in a dose-independent manner^43^. It was reported that psoromic acid has antioxidant and cardiovascular protective effects^44^. These top differential metabolites have their own effects and they work together to change the pharmacological activity of *Salvia miltiorrhiza*.

### Newly generated and disappeared compounds

**Figure 6:**
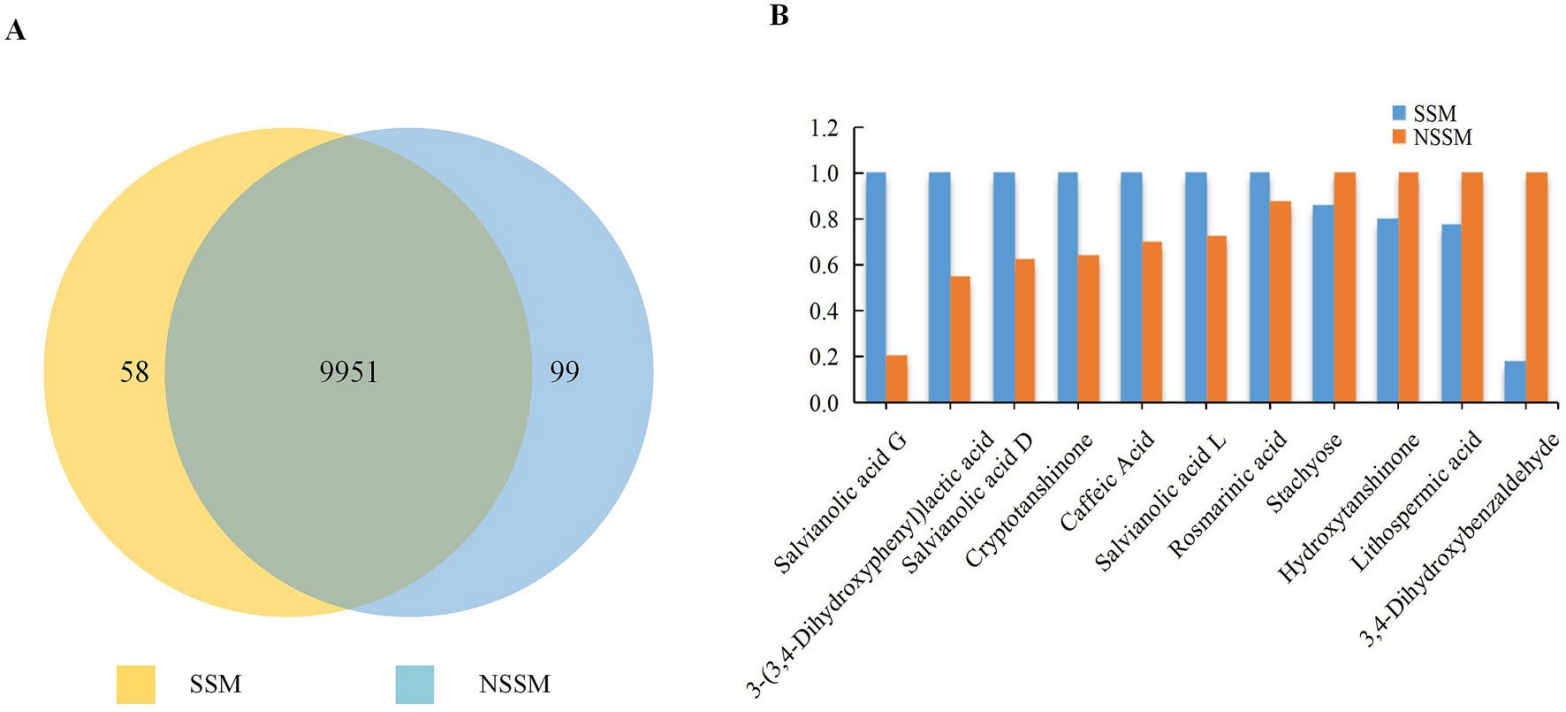
Comparison of differential and characteristic metabolites. **a** Venn diagram shows newly formed and vanished compounds after “*Sweating*”, **b** the content of differential characteristic metabolites in SSM and NSSM (the contents of each higher one covert to 1.0).

By comparing the sum of fragments ion strength of each substance peak, 58 substance peaks only emerged in SSM, among which 31 metabolites identified, including 19 from positive ion models (ESI^+^) and 12 from negative ion models (ESI^-^). 99 substance peaks were exclusively determined in NSSM with 49 metabolites identified, which contained 27 and 22 differential metabolites from ESI^+^ and ESI^-^, respectively (Fig. 6a, Supplementary Table 5). Among these 80 identified (31 in SSM and 49 in NSSM) exclusive metabolites, 20, 9, 6, 4, 4, 3 of them belonged to carboxylic acids and derivatives, polyketides, fatty acyls, flavonoids, prenol lipids, and benzene and substituted derivatives, respectively.

### Characteristic Metabolites analysis by “*Sweating*”

Tanshinones and Salvianolic acids are the two kinds of characteristic bioactive compounds possessing magnificent bioactivities in Danshen. In this untargeted metabolomic study, a total of 23 specific compounds of Tanshinones and Salvianolic acids had been detected (Supplementary Table 6), and there was significant difference in content of 11 of them. And among these 11 characteristic differential metabolites, 7 metabolites content increased while 4 decreased in SSM compared with NSSM. In addition, there was the largest increase in the Salvianolic acid G content while the greatest reduction in the 3, 4-Dihydroxybenzaldehyde content of SSM compared to NSSM. (Fig. 6b). Correlating our previous study on Chromatographic Fingerprint of 10 target characteristic metabolites^45^, we can come to the conclusion that there was a change in most of Tanshinones and Salvianolic acids content after *“Sweating”*. We suggest that a transcriptomic (We had confirmed there was no degradation in total RNA after “*Sweating*”) or a proteomic study should be performed to investigate the change in genes or enzymes of Danshen during “*Sweating*” processing to explain the change of metabolites content of SSM.

## Conclusions

Origin processing of “*Sweating*” has a significant effect on the metabolites content and composition of *S*. *miltiorrhiza*. There are 287 differential metabolites between sweated and non-sweated *S*. *miltiorrhiza* with 112 up-regulated and 175 down-regulated, and they mainly belong to lipids and lipid-like molecules, phenylpropanoid and polyketides, and Benzenoids. And these differential metabolites are mainly enriched in the pathway of linoleic acid metabolism, glyoxylate and dicarboxylate metabolism and phenylpropanoid biosynthesis. Total of 80 metabolites exclusively detected mainly belong to carboxylic acids and derivatives, polyketides, fatty acyls and flavonoids. Among them, 31 and 49 are detected in SSM and NSSM, respectively. A total of 23 metabolites that belong to tanshinones and salvianolic acids had been identified, and 11 metabolites content was changed significantly after *“Sweating”.*

## Materials and methods

### Chemicals and plant materials

Plants of *S*. *miltiorrhiza* Bunge were cultivated in National Breeding Base of Seeds and Seedlings of Medical Herbs for Essential Drugs in Anhui University of Chinese Medicine, Hefei, China.

Methanol, formic acid, water for LC/MS analysis and acetonitrile were all purchased from CNW Technologies GmbH (Düsseldorf, Germany). Lmurine-2-chlorophenylalanine was obtained from Shanghai Hengchuang Bio-technology Co., Ltd. (Shanghai, China). LysoPC17: 0 was purchased from Avanti Company of the United States, and all of the chemicals and solvents were analytical or HPLC grade.

### “*Sweating*” processing of plant materials

The same batch of *S. miltiorrhiza* was with all kinds of size collected, washed thoroughly and put in an open, clean, dry and cool place, so as to avoid overstocking during the process of natural water loss, preventing heat production. Then, reed heads were removed from the roots and picked over ones with uniform thickness and randomly divided them into two groups. One group (SSM) was processed by “*Sweating*”, and the other group (NSSM) was unprocessed and directly stored at – 80 ℃. The steps of “*Sweating*” processing which are screened by modifying report from Liang et al. are as follows^46^: More than 100 kg fresh roots with different sizes were piled up for seven days and covered with a sack to produce heat so as to make the internal moisture of root tissues spread outward in an open place. In order to make the whole roots sweat evenly, the roots were spread out for a night and then covered with sack every two days. When the internal sections of the roots turned purplish red, end “*Sweating*” and gather them up. Six independent sample pairs were collected from two groups and each sample pair of the same size were ground into powder after being lyophilized in a speed vacuum and sieving with 50 order mesh were stored at − 80 ℃.

### Sample preparation

The powdery samples (80 mg) after being precisely weighed were transferred to 1.5 mL Eppendorf tubes, and two small steel balls were added to each tube. Two internal standards 17:0 Lyso PC (1-heptadecanoyl-sn-glycero-3-phosphocholine), 0.01 mg/mL; (20 μL each) (L-2-chlorophenylalanine), 0.3 mg/ mL, both of them were prepared with methanol and 1 mL mixture of methanol and water (3/7, v/v) were added to each sample, and the samples were pre-cooled at − 20 ℃ for 2 min. Then the samples were ground at 60 Hz for 2 min, and stood under ultrasonication at ambient temperature for 30 min, and placed at − 20 ℃ in refrigerator for 20 min. Samples were centrifuged at a speed of 13,000 rpm, 4 °C for 10 min. 300 μL of supernatant in a brown glass vial was dried in a freeze concentration centrifugal dryer, and then added 400 μL mixture of methanol and water (1/4, v/v) to each sample, vortex for 30 s, and ultrasonicated for 2 min. Samples were centrifuged again at 13,000 rpm, 4 ℃ for 10 min. The supernatants (about 150 μL) from each tube were collected using crystal syringes, filtered through microfilters at 0.22 μm and transferred to LC vials, and stored at − 80 °C for LC–MS analysis. QC samples were prepared by mixing aliquots of 6 SSM and 6 NSSM to be a pooled sample, and used to balance the chromatography-mass spectrometer system.

### LC/MS-based metabolomics analysis

A Dionex Ultimate 3000 RS UHPLC system fitted with Q-Exactive quadrupole-Orbitrap mass spectrometer equipped with heated electro-spray ionization^47^ source (Thermo Fisher Scientific, Waltham, MA, USA) was used to analyze the metabolic profiling in both ESI positive and ESI negative ion modes. An ACQUITY UPLC BEH C18 column (1.7 μm, 2.1 × 100 mm) was employed in detecting both positive and negative modes. The binary gradient elution system consisted of (A) 0.1% (v/v) formic acid dissolved in water and (B) 0.1% formic acid (v/v) dissolved in acetonitrile and separation was achieved using the following gradient: 0 min, 5% B; 1.5 min, 5% B; 3 min, 30% B; 7 min, 60% B; 9 min, 90% B; 11 min, 100% B; 13 min, 100% B; 13.2 min, 5% B; 16 min, 5% B. The flow rate was at 0.35 mL/min and column temperature was at 40 ℃. All the samples were kept at 4 ℃ during the detection, the injection volume was at 5 μL each time, and the mass range was from m/z 100 to 1000. The resolution was set at 70,000 for the full MS scans and 17,500 for HCD MS/MS scans, and the Collision energy was set at 10, 20 and 40 eV. The mass spectrometer operated as follows: spray voltage, 3500 V at negative and positive mode; capillary temperature, 320 ℃; sheath gas flow rate, 40 arbitrary units; auxiliary gas flow rate, 10 arbitrary units.

The QCs were injected at regular intervals (every 6 samples) throughout the analytical run to provide a set of data from which repeatability can be assessed, and were used to evaluate the stability of the mass spectrometer system during samples detection.

### Data reduction and multivariate data analysis

The acquired LC–MS raw data were filtered by the software of Progenesis QI v2.3 (Nonlinear Dynamics, Newcastle, UK) through steps of baseline filtering, peak recognition, integration, retention time correction, peak alignment and normalization, and using the following parameters. Precursor tolerance was set at 5 ppm, product tolerance was set at 10 ppm, product ion threshold was set at 5%, and retention time (RT) tolerance was set at 0.02 min. Internal standard detection parameters were deselected for peak RT alignment, isotopic peaks were excluded for analysis, and noise elimination level was set at 10.00, minimum intensity was set to 15% of base peak intensity. The Excel file was obtained with three dimensions data sets including m/z (mass-to-charge ratio), peak RT (retention time) and peak intensities, and RT–m/z pairs were used as the identifier for each ion. The internal standard was used for data QC (reproducibility)^48^.

Metabolites were identified by the Progenesis QI Data Processing Software (version 2.3, https://www.nonlinear.com/progenesis/qi/)^49^ based on precise mass number, secondary fragments and isotope distribution, and qualitative by mapping on the Human Metabolome Database (HMDB), Lipidmaps (v 2.3) and METLIN database^50^.

For the extracted data, delete the ion peaks whose missing values (ion intensity  = 0) is more than 50% in the group, and replace the 0 value with the half of the minimum value, and screen the qualitative compounds according to the score of the qualitative results of the compounds. The screening standard is 30 points (Full score 60 points), and if the score is lower than 30 points, the qualitative results are deemed to be inaccurate and deleted. Finally, the positive and negative data were combined to get a combined data matrix table which contained all the information extracted from the original data, and was imported into R ropls package. Principle component analysis (PCA) and (orthogonal) partial least-squares-discriminant analysis (O)PLS-DA were carried out to visualize the metabolic alterations between sweating and non-sweating groups, after mean centering (Ctr) and Pareto variance^51^ scaling, respectively. The Hotelling’s T2 region, shown as an ellipse in score plots of the models, defines the 95% confidence interval of the modeled variation. Variable importance in the projection (VIP) ranks the overall contribution of each variable to the OPLS-DA model, and those variables with VIP ≥ 1.0 are considered relevant for group discrimination^52^.

In this study, the default 7-round cross-validation and 200 times of response permutation testing (RPT) was applied with 1/seventh of the samples being excluded from the mathematical model in each round, in order to review the model quality and guard against over-fitting. The differential metabolites were selected on the basis of the combination of a statistically significant threshold of variable influence on projection (VIP) values obtained from the OPLS-DA model, P values from a two-tailed Student’s t test on the normalized peak areas and the adjusting P value for correcting was obtained by Benjamini & Hochberg method, and fold change (FC) value SSM verse NSSM, where metabolites with VIP values ≥ 1.0 and p values < 0.05 were considered as differential metabolites. While calculating FC values, if the datum displaying the content of metabolite is zero, it would be changed by the half of the minimal corresponding value in the table of data matrix (2.5E-06)^48^.

### Univariate statistical analysis and differential metabolites visualization

The experimental data obtained in the present study is expressed as the mean ± standard deviation (SD) of six independent biological replicates, and the significance level of the metabolites between groups was calculated by one-way analysis of variancewith Student’s t test and fold change analysis using GraphPad Prism 7.00 software^53^.

The volcano plot was used to visualize the P value from student’s t test and the fold change (FC) value, which is conducive to screening differential metabolites, as shown in the figure with three different colors (red, blue and gray) to represent significantly up-regulated, down-regulated and non-significant differential metabolites in the experimental group.

### KEGG pathway analysis of differential metabolites

Mapping the differential metabolites to KEGG data to look for their KEGG ID and the pathway they belonged to, then counting the number of metabolites enriched in corresponding pathway. The -lg(*P*-value) was used to measure whether the pathway enriched or not, and the pathway was considered as enriched pathway when *P*-value ≤ 0.05^50^.

$$ p = 1 - \sum\limits_{i = 0}^{m - 1} {\frac{{\left( {\mathop {\text{M}}\limits_{{{\rm i}}} } \right)\left( {\mathop {{\text{N}} - {\text{M}}}\limits_{{{{\rm n}} - {\text{i}}}} } \right)}}{{\left( {\mathop {\text{m}}\limits_{{{\rm n}}} } \right)}}} $$

N: The number of total metabolites, n: The number of differential metabolites, M: Number of metabolites in specific pathway, m: Number of differential metabolites in specific pathway.

## Supplementary information

**Figure 1** Hierarchical Clustering of Expression Quantity of Identified Metabolites.

**Table 1** Data matrix of metabolomic analysis.

**Table 2** Differential metabolites between SSM and NSSM.

**Table 3** KEGG pathway enrichment data.

**Table 4** Top 50 differential metabolites from metabolomic analysis.

**Table 5** Extreme variant substance peaks and metabolites.

**Table 6** Specific Metabolites of tanshinones and salvianolic acids.

## Data Availability

The datasets generated during the current study are available in the Baidu Cloud repository: https://pan.baidu.com/s/169-IDR72hzC6CvS0b537fA. Access code: bb71.
