# Supplementary figures and images for: UPLC/MS-based untargeted metabolomics reveals the changes of metabolites profile of Salvia miltiorrhiza bunge during Sweating processing
Source: Sci Rep. 2020 Nov 11;10:19524. doi: 10.1038/s41598-020-76650-w (PMC7658355; doi:10.1038/s41598-020-76650-w)

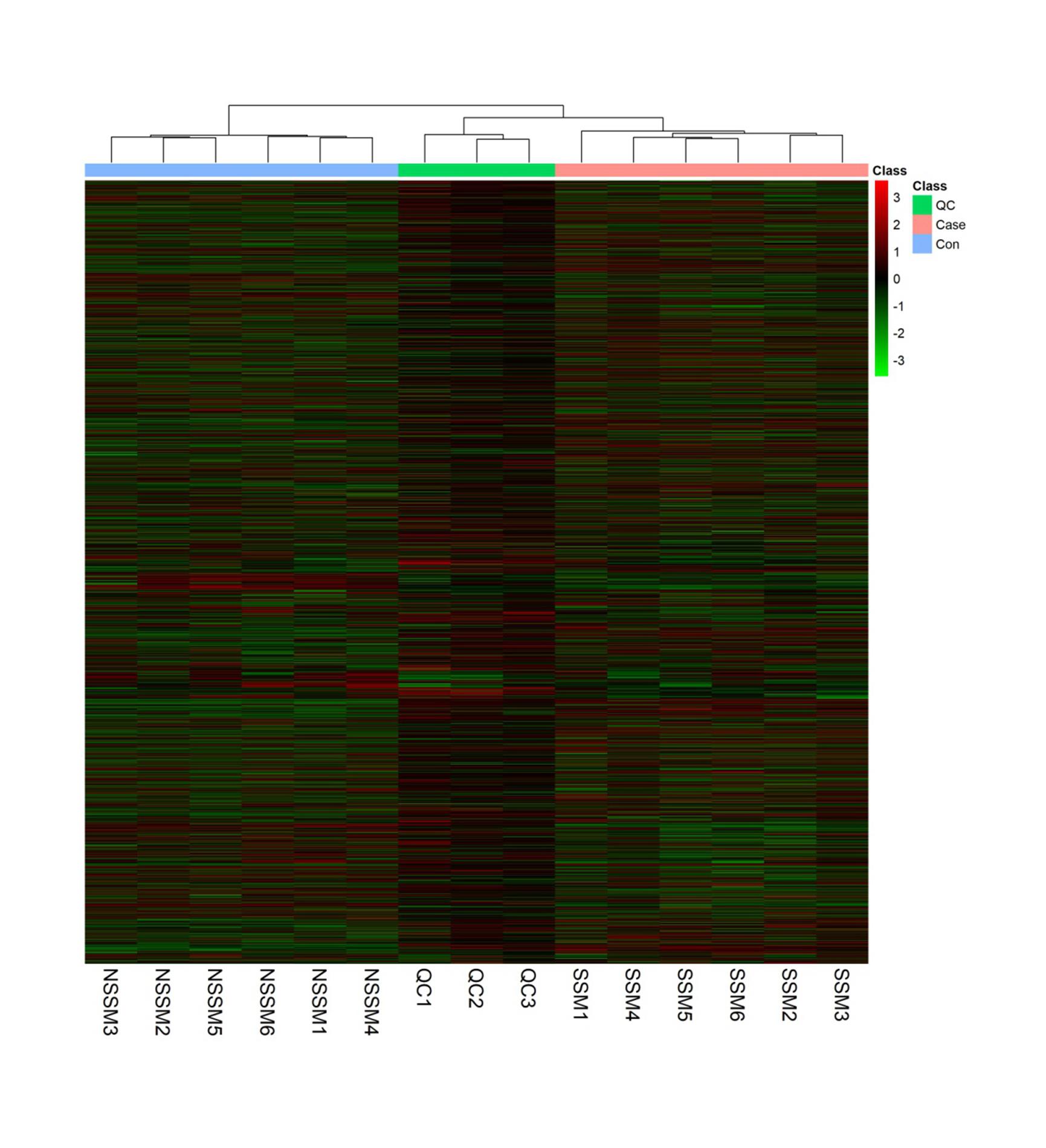

Supplement: Supplementary file 1 — Figure 1 Hierarchical Clustering of Expression Quantity of Identified Metabolites. [file 41598_2020_76650_MOESM1_ESM.doc]
